# Can anatomy-based fitting improve musical perception in adult cochlear implant users?

**DOI:** 10.1016/j.bjorl.2024.101533

**Published:** 2024-11-19

**Authors:** Luis Lassaletta, Miryam Calvino, Isabel Sánchez-Cuadrado, Elena Muñoz, Javier Gavilán

**Affiliations:** a Hospital Universitario La Paz, IdiPAZ Research Institute, Department of Otorhinolaryngology, Madrid, Spain; b Biomedical Research Networking Centre on Rare Diseases (CIBERER), Institute of Health Carlos III, Madrid, Spain; c Universidad Autónoma de Madrid, Spain; d MED-EL Elektromedizinische Geräte GmbH, Department of Clinical Engineering, Madrid, Spain

**Keywords:** Cochlear implant, Music, Anatomy-based fitting, Outcomes, Questionnaires

## Abstract

**Objectives:**

To compare Anatomy-Based Fitting (ABF) vs. Default Fitting (DF) in terms of music perception and music enjoyment in CI users.

**Methods:**

CI users ≥ 18 years implanted with a Synchrony ST Flex28 (MEDEL) with at least 12 months of experience with either ABF or DF, with good speech tests outcomes (≥ 65% disyllables) were enrolled. Music tasks were assessed with “Discovery” level of Meludia online interactive music tool, which includes Rhythm (beat count), Spatialization (differentiating between lower and higher notes), Stable/unstable (perception of stability or instability), Melody (ascendant or descendant), and Density (presence of one or multiple simultaneous sounds). Each task consists of five levels of difficulty. Participants were allowed a maximum of 4 attempts to complete a level. The Music-Related Quality of Life (MuRQoL) and the Munich Music (MUMU) questionnaires evaluated music background.

**Results:**

Twenty CI users took part: 10 using ABF and 10 DF. There were no significant differences in age at implantation (53.2 ± 15.0 vs. 53.8 ± 14.5y), time since surgery (1.8 ± 0.5 vs. 2.4 ± 1.5y), or the percentage of disyllables in silence (68.0 ± 8.0 vs. 77.3 ± 11.5%) between groups. Regarding Meludia scores, differences were observed between both groups in Density: ABF had better mean scores when completing the level in only one attempt (8.7 ± 2.5 vs. 4.6 ± 3.1, *p* = 0.016) and required fewer restarts to complete the five levels (6.6 ± 1.6 vs. 9.3 ± 2.3; *p* = 0.009). Furthermore, the fifth level of Density was successfully completed by 60% of patients with ABF, while none of the patients with DF achieved this (*p* = 0.003). The scores obtained in MuRQoL were similar between ABF and DF groups. In a similar way, the MUMU showed no differences in terms of frequency and music importance.

**Conclusions:**

ABF demonstrates specific advantages in certain musical tasks, specifically in processing multiple sounds simultaneously. Further studies are necessary to explore additional benefits of ABF in musical skills in CI users. NCT05319678.

**Level of evidence:**

Level 3.

## Highlights

• Music represents one of the biggest challenges for cochlear implant users.
• Anatomy-based fitting is a new step regarding personalized medicine in the field of CIs.
• Anatomy-based fitting may be beneficial for improving certain musical skills in CI users

## Introduction

Cochlear Implants (CIs) have emerged as a global solution for individuals experiencing severe to profound deafness, promoting effective speech discrimination and significantly enhancing their quality of life.^1^ However, outcomes vary widely among users. While many implantees achieve high levels of speech discrimination, challenges persist in areas such as understanding speech in noisy environments or enjoying music fully. On one hand, music perception and enjoyment with a CI depend on the CI performance. As a general rule, CI users often have difficulty faithfully replicating the intricate harmonic structures and timbres found in music.^2^, ^3^ The electrical stimulation from a CI lacks the precision of the cochlea's natural processing, resulting in a musical experience that is less rich and lacks the subtle nuances. On the other hand, both music perception and enjoyment are also influenced by subjective factors like musical background and personal interest, adding layers of complexity to the goal.^4^, ^5^

In recent years, personalized medicine has begun to influence the field of CIs. There is increasing interest in measuring the cochlear duct length, choosing an appropriate electrode array, and aiming for deep cochlear insertion to stimulate lower frequencies. This is vital for both noise discrimination and music perception. This approach has led to what is known as frequency-based, imaging-based, or Anatomy-Based Fitting (ABF). This fitting method aims to reduce the mismatch that exists between the real tonotopic (anatomic) frequency, and the frequency assigned to the electrode at the same tonotopic location by a default fitting (DF). In recent years, several studies have analyzed the possible benefit of ABF, most studies focusing on speech perception.^6^, ^7^ While the actual advantages of this fitting method are currently under investigation, theoretically, electrical stimulation with a CI that better aligns with the anatomical frequency distribution should lead to improved CI performance^8^ and consequently, better music perception.

The present study compared music perception using the Meludia music platform^9^ and music enjoyment using the Music-Related Quality of Life (MuRQoL) questionnaire and Munich Music (MUMU) questionnaire between two groups of post lingually deaf CI users unilaterally implanted, the first group with a DF, and the second one with an ABF.

## Methods

### Participants

This study was approved by the local Ethics Committee (approval number HULP PI-4447) and was registered at ClinicalTrials.gov (identifier NCT05319678).

This was a cross-sectional study carried out between April 2022 and November 2023 at La Paz University Hospital (Madrid, Spain).

Two groups of subjects were recruited: CI users fitted with ABF and CI users with default (logarithmic) fitting.

They were post lingually deaf unilateral CI users ≥18 years implanted for a first time with a Synchrony ST Flex28 electrode array (MED-EL, Innsbruck, Austria).

Moreover, they must have at least 12 months of stable fitting (time by which they should have reached a plateau in speech recognition),^10^ had at least 10 active electrodes and good speech tests outcomes (≥65% disyllables identification in silence).

All participants were fluent in Spanish and were without concomitant visual or cognitive impairments that could interfere with completion of the musical tasks. All of them had no prior formal musical training.

### ABF and DF procedures

As previously described by our team^7^ postoperative computed tomography data were used for ABF fittings. Briefly, cochlear parameters were determined using the clinical surgical planning software OTOPLAN v3 (CAScination, AG, Bern, Switzerland), which allows the localization of the angular position and tonotopic frequency of each electrode contact in each individual cochlea. These parameters were then exported to the fitting software MAESTRO v9.0 from MEDEL, and a new frequency map was elaborated.

Furthermore, in DF the fitting software MAESTRO v9.0 provides default frequency filters distributed across the active electrodes of the electrode array.

If an electrode had to be switched off for any reason, the remaining frequency bands were redistributed among the other channels. The coding strategy used in both fittings (DF and ABF) was FS4-P.

### Evaluation of music perception: Meludia

**Fig. 1:**
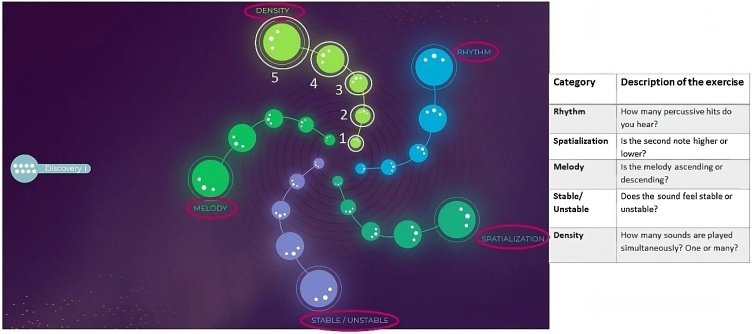
Meludia’s interface. The “Discovery” module with its five categories (Density, Rhythm, Spatialization, Stable/unstable, Melody), and a description of them are shown. Modified from www.meludia.com.^9^

Meludia is a web-based music training software based on the different music dimensions.^9^ For this study, the Discovery module was used. This module includes five categories with five levels of difficulty (total: 25 exercises) (Fig. 1). Fig. 1 also shows a description of the task associated with each category.

How to proceed with the music software was explained to all the participants in a quiet room before the evaluation. CI users used the Direct Audio Input (DAI) cable to ensure that hearing was tested via CI alone.

The numeric scores of Meludia were rated from 0 to 3, where 0 indicates a no complete attempt and 3 indicates a fast a precise carrying out of the exercise.

A maximum of four restarts for each level was allowed. If the exercise was not completed after four restarts, it was considered as incomplete and the listener would change to the next category, the score in the incomplete level being zero. The evaluation starts with the “Rhythm” category.

The Meludia test was evaluated in three categories: (i) Mean score obtained in each category when the level is completed in only one attempt; (ii) Total number of attempts to achieve the last category, and (iii) If the last (fifth) level was completed or not (for more details, please refer to^11^ and^12^).

### Evaluation of music background. Questionnaires

To test musical background, two questionnaires were used: the MuRQoL questionnaire and the MUMU Questionnaire.

#### (MuRQoL) questionnaire

The MuRQoL uses a 5-point Likert-type scale to assess frequency and importance items related to music.^5^ The Spanish version, recently validated by our group,^12^ was used. It consists of two parts, each of them with 18 questions, e.g.: “Can you recognize the words in songs?”, “How important is it for you to be able to recognize the words in songs?” (Overall scale). Moreover, the questions in each part are divided into perception and engagement subscales.

#### MUMU questionnaire

The MUMU translated and adapted to Spanish was used to assess the music listening habits of post lingually CI users.^13^ The original version contains 25 questions to register these music listening habits in relation to several instruments, musical styles, how music sounds with the CI, etc. For this study we used a simplified version to limit the duration of each evaluation (as others did previously^14^), which contains the questions number 1, 2, 4, 7, 8, 13 and 16 of the original version. We considered these questions were more relevant to the purpose of our study. The options of responses were scales of classification and multiple-choice questions.

### Statistical analysis

Demographic characteristics and outcome measures are shown as absolute (n) and relative (%) frequencies and if appropriate as mean plus Standard Deviation (±SD) and range.

Each Meludia level was scored as complete or incomplete. The mean ± SD was also calculated.

Intergroup (patients with ABF and patients with DF) comparison of scores was evaluated via U-Mann Whitney test. Chi-Square was used to compare the number of participants who completed the levels of each Meludia category.

Spearman’s correlation coefficient was calculated to assess the relationship between mean score of the different categories of Meludia program, and the corresponding subscales of MuRQoL and MUMU scores.

Missing data were treated as missing values. A level of p ≤ 0.05 (2-tailed) was considered significant. Statistical analyses were processed in SPSS software package version 24.0 (IBM, Armonik, NY, USA).

## Results

### Group characteristics

Twenty CI users participated in the study: 10 using ABF and 10 with DF. Demographic and hearing characteristics of both groups are displayed in Table 1. Both groups were similar in terms of gender, age, hearing loss features and use of their CI.

**Table 1:** Demographic characteristics and speech discrimination outcomes of the patients included in the study.

	ABF	DF	p (*U*-Mann Whitney)
n	10	10	
Male (n) (%)	3 (30%)	3 (30%)	
Female (n) (%)	7 (70%)	7 (70%)	
Formal education (years) (mean ± SD) (range)	13.9 ± 3.4 (10–20)	12.9 ± 2.8 (6–17)	0.631
Age at implantation (years) (mean ± SD) (range)	53.2 ± 15.0 (28–73)	53.8 ± 14.5 (26–78)	0.853
Duration of hearing loss (years) (mean ± SD) (range)	22.4 ± 12.0 (8–46)	28.7 ± 16.7 (0–54)	0.297
CI use (years) (mean ± SD) (range)	1.8 ± 0.5 (1–3)	2.4 ± 1.5 (1–5)	0.529
Etiology, n (%)			
Unknown	7 (70%)	6 (60%)	
Otoesclerosis	0	3 (30%)	
Ototoxic	1 (10%)	0	
Chronic otitis media	1 (10%)	0	
Sind. Meniere	0	1 (10%)	
Sudden hearing loss	1 (10%)	0	
Hearing condition, n (%)			
CI alone	5 (50%)	4 (40%)	
Bimodal	5 (50%)	6 (60%)	
Disyllables in silence (%) (mean ± SD) (range)	68.0 ± 8.0 (58–78)	77.3 ± 11.5 (58–92)	0.079

SD, Standard Deviation.

### Performance on Meludia tasks

Regarding the mean score obtained in each category when the level is completed in only one attempt, patients with ABF had better Density scores than those with DF (8.7 ± 2.5 vs. 4.6 ± 3.1, *p* = 0.016) No differences in the other tasks were found between both groups (Table 2).

**Table 2:** Meludia scores.

	Rhythm			Spatialization			Stable/unstable				Melody				Density		
	Meludia scores, only one attempt	Sum of all attempts	Percentage of CI users who completed the 5^th^ level	Meludia scores, only one attempt	Sum of all attempts	Percentage of CI users who completed the 5^th^ level	Meludia scores, only one attempt	Sum of all attempts	Percentage of CI users who completed the 5^th^ level	Meludia scores, only one attempt		Sum of all attempts	Percentage of CI users who completed the 5th level	Meludia scores, only one attempt		Sum of all attempts	Percentage of CI users who completed the 5^th^ level
ABF	13.6 ± 1.3	5.2 ± 0.6	80%	13.5 ± 9.6	6.3 ± 1.2	60%	9.0 ± 0.8	8.0 ± 3.1	40%	12.0 ± 0.0^a^		7.9 ± 1.9	10%	8.7 ± 2.5		6.6 ± 1.6	60%
DF	12.1 ± 4.1	6.2 ± 2.0	80%	9.6 ± 5.4	5.6 ± 1.6	70%	4.0 ± 5.1	6.3 ± 2.0	20%	5.0 ± 3.8		7.8 ± 1.9	10%	4.6 ± 3.1		9.3 ± 2.3	0%
*U-*Mann Whitney / χ^2^	0.460	0.393	1.000	0.073	0.165	0.639	0.106	0.218	0.329	0.182		0.853	1.000	0.016^b^		0.009^b^	0.003^b^

First column of each task: Scores of each category when the level is completed in only one attempt.

Second column of each task: Sum of all the attempts.

Third column of each task: Percentage of CI users who completed the 5th level.

Data shown are mean ± standard deviation.

a Note that only one patient (ABF) completed the five levels of melody without restarts.

b Means *p* ≤ 0.05.

Considering the number of attempts to achieve the last level of each task, ABF patients needed less attempts to complete the five Density levels (6.6 ± 1.6 vs. 9.3 ± 2.3, *p* =  0.009) compared to subjects with DF (Table 2).

When evaluating if the last (fifth) level of each task was completed or not, no patients with DF completed the 5th level of Density task, where more than half of ABF patients (60%) completed it (*p* = 0.003). Table 2 shows the percentage of patients who finished the last level (5th level) of each category.

### Questionnaires

#### MuRQoL

**Fig. 2:**
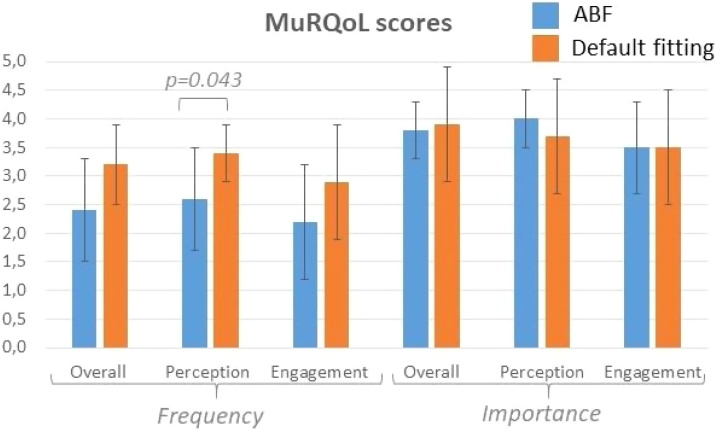
MuRQoL scores of both groups. Overall, perception and engagement scores of the frequency and importance part were displayed.

Fig. 2 shows the scores obtained both in the overall scales and in the perception and engagement subscales of the MuRQoL questionnaire. No significant difference was found in the overall scores (both in the frequency and importance sections), but in the perception subscale of the frequency part where subjects with ABF scored slightly lower than those with DF (*p* = 0.043).

#### MUMU

Regarding the MUMU questionnaire scores before and after cochlear implantation in terms of frequency and music importance, no differences were found between both groups (Table 3).

**Table 3:** Mean ± standard deviation (from a 10-point scale) of Questions (Q) 1, 2, 3 and 4 of MUMU questionnaire and the mean scores of the differences between those topics before and after cochlear implantation.

	How often did you listen to music before your hearing loss? (Q1)	How often do you listen to music now, after receiving your CI? (Q2)	What role does music play in your life before the onset of your hearing loss? (Q3)	What role does music play in your life now, since receiving your CI? (Q4)	Score of the difference between frequency of listening to music before and after the CI (Q1‒Q2)	Score of the difference between the role of music in their life before and after the CI (Q3‒Q4)
ABF	7.8 ± 2.3	3.7 ± 2.6	6.1 ± 3.0	3.2 ± 2.2	4.1 ± 3.7	2.9 ± 4.1
Default fitting	7.6 ± 2.7	5.4 ± 3.0	8.1 ± 1.9	5.7 ± 2.9	2.2 ± 5.0	2.4 ± 3.8
*U*-Mann Whitney	0.968	0.356	0.113	0.079	0.497	0.842

## Discussion

### Main results

The aim of this study was to determine the benefit of ABF in music perception and enjoyment. We compared the Meludia music test and two subjective music questionnaires in two similar groups of bilaterally deaf unilateral CI users, one with a default CI fitting and the other with an ABF. In this study patients with ABF achieved better Density scores and needed less restarts to complete the last level of the Density task than those with DF. No significant differences were found in other music tasks or in the subjective music questionnaires.

### Music as a goal for CI users-difficulties. How to improve music perception in CI users

As the CI current technology does not provide enough of the temporal cues for accurate pitch and timbre perception, music is usually described as unpleasant for many CI users.^15^ Apart from the differences in musical background or personal preferences, detecting changes in pitch or recognizing different instruments or voices in daily music is really challenging for most implantees.^16^, ^17^ Most CI users would like to improve music perception. This has been proposed to overcome in several ways.

#### Simplify or change the music stimuli

Some authors have proposed to simplify or change the music stimuli in order to make it more accessible to CI users.^18^ Althoff et al.^19^ analyzed potential modifications to instrumental music aimed at enhancing accessibility for CI users. These authors found that CI users favored an increased volume on the percussion component, which delivered a clear musical rhythm and tempo. Additionally, they generally preferred all instruments to be panned to their preferred side, typically the right side, with the percussion component being the most prominent. Other authors have used signal processing techniques in order to customize music to make it more appealing for CI users.^20^, ^21^

Hwa et al.^22^ showed an increase in music enjoyment for CI users with a patient-directed music re-engineering using a web-based audio mixer. The CI users showed a preference for increased bass, reverberation, and treble across various musical genres.

#### Add vocals or vibrotactile info to the melodies

It is well-known that recognition of songs by CI users is usually based on the lyrics. By incorporating vocals, CI users may find it easier to perceive and enjoy the various elements of music, leading to a more fulfilling auditory experience.^16^ Recently, our group has concluded in a study carried out in children that CI users were significantly worse at recognizing instrumental music than their normal hearing peers.^4^ Moreover Verma et al.^23^ have investigated how vibrotactile stimulation affects timbre perception in both normal-hearing listeners and CI users. In their study, tactile stimuli affected timbre perception in both groups. However, in normal-hearing listeners' similarity judgments were primarily driven by auditory stimuli, whereas CI users placed more emphasis on the tactile stimuli. The authors propose that future devices could use vibrotactile stimulation to enhance timbre perception cues for CI users.

#### Music training

As there are no established guidelines for auditory rehabilitation following cochlear implantation,^24^ using music as a routinely rehabilitation method is still relatively new. Research by several authors has demonstrated that both training and exposure can significantly enhance music perception and appreciation.^2^, ^25^, ^26^ In 2022 Boyer and Stohl^27^ described the use of the Meludia music platform as a music training program with progressive listening exercises to train music perception in adult CI users. Calvino et al.^11^ showed its usefulness in children and adolescents. Veltman et al.^28^ have recently detailed a music training program (Musi-CI) aimed at overcoming the aversion that some post-lingually deaf CI users experience when listening to music.

#### Modify fitting of audio-processors

Another approach to improve music perception is to modify the fitting in order to make all the music components more accessible to CI users. Tahmasebi et al.^29^ decreased the number of bands used for stimulation, improved noise reduction to minimize background instrument levels, and utilized back-end amplitude compression to enhance the contrast of lyrics in music with vocals.

In recent years, the new concept of tonotopic, imaging, or ABF has been tested to demonstrate its benefits in various scenarios, including music perception. The goal of this fitting method is to align the frequency map of the CI with the tonotopic frequency map of the cochlea. This process involves measuring the cochlear duct length and the electrode positions using specialized planning software. These cochlear measurements are then imported into the fitting software to create a frequency-band distribution that better matches the tonotopic frequency distribution.^7^

### ABF and speech discrimination

The concept of ABF has been evaluated in various clinical scenarios, showing both subjective and objective improvements over the DF method. Generally, the benefits of ABF are more pronounced when it is performed on one side while the patient retains some degree of residual hearing on the other side. This alignment of frequency distribution in both ears is particularly beneficial for single-sided deafness or bimodal users with a good hearing aid performance.^6^, ^30^

In bilaterally deaf patients with a unilateral CI, objective improvements in speech discrimination may be less pronounced. However, most patients demonstrate a subjective preference for ABF compared to DF.^7^ Despite this, the majority of CI candidates fall within this population, and it is crucial to make every effort to enhance performance for these individuals.

### ABF and music

Theoretically, a better matching between electrical stimulation with a CI and the anatomical tonotopic frequency distribution should lead to an improvement not only in speech discrimination but also in music perception and thus enjoyment. Heitkotter et al.^31^ analyzed if deeper insertions leading to a lower frequency mismatch had an impact on perception of musical attributes using the Montreal Battery of Evaluation of Amusia (MBEA test). They found no effect of increased electrode depth on better detection and identification of pitch and tonality. The authors suggest that ABF may be able to improve these tasks.

### ABF and music perception

In the present study, patients with ABF achieved better results in the Density task of the Meludia platform, both in scores and in the attempts needed to complete all the levels of the task. Interestingly, no patients with DF completed the 5th level of Density task, whereas almost two thirds of the ABF patients did. According to the Meludia program, our brain naturally tends to focus on higher-pitched sounds when multiple notes are played simultaneously, potentially struggling to distinguish middle and lower sounds.^9^ It has been suggested that CI users manage better the perception of chord sequences than melody recognition.^32^ However, with training, the ability to perceive and process multiple sounds simultaneously can be enhanced, leading to notable advancements in musical understanding. Initially, subjects are prompted to identify whether they perceive a single note or multiple notes, marking the initial and fundamental step in recognizing the composition of chords. Our results suggest ABF to provide specific musical advantages in processing multiple sounds.

### ABF and subjective music enjoyment

The benefit ABF on subjective music enjoyment was recently assessed by Fan et al.^33^ The study compared two groups of Mandarin-speaking CI adult users with postlingual deafness: one group received anatomic mapping frequency reallocation (n = 25), while the other group received default frequency distribution (n = 23). In addition to observing improved speech discrimination in noise in the ABF group, results from the MUMU questionnaire indicated that patients showed a stronger inclination to engage with music following the procedure involving frequency reallocation compared to the default setting. In the present study, no significant differences were found between the ABF and DF groups in either the MUMU or MuRQoL scores. A slight tendency was found towards better scores in subjects with DF, which may be explained by musical background and personal interest may, but also to the study design and/or the limited sample size in each group.

## Conclusions

In the present study ABF demonstrated specific musical advantages in processing multiple sounds simultaneously; that is, the Density Meludia task. To our knowledge this is the first study evaluating music perception and enjoyment, both with objective testing (Meludia) and subjective music questionnaires (MUMU and MuRQoL). Further studies are necessary to explore additional benefits of ABF in musical skills.

## Funding

This manuscript has no prior publication and was partially supported by a grant (PI21/00147) from Programa Estatal de Generación de Conocimiento y Fortalecimiento del Sistema Español de I+D+I, ISCiii, Spain.

There is no reproduction of pre-published information/material in this article.

## Declaration of competing interest

Elena Muñoz is employed at MED-EL as a clinical engineer; this function entails support during fitting sessions. MED-EL was not involved in the study design, collection and analysis of data or the decision to submit it for publication.
